# Sequence-based multiscale modeling for high-throughput chromosome conformation capture (Hi-C) data analysis

**DOI:** 10.1371/journal.pone.0191899

**Published:** 2018-02-06

**Authors:** Kelin Xia

**Affiliations:** 1 Division of Mathematical Sciences, School of Physical and Mathematical Sciences, Nanyang Technological University, Singapore 637371, Singapore; 2 School of Biological Sciences, Nanyang Technological University, Singapore 637371, Singapore; Emory University Rollins School of Public Health, UNITED STATES

## Abstract

In this paper, we introduce sequence-based multiscale modeling for biomolecular data analysis. We employ spectral clustering method in our modeling and reveal the difference between sequence-based global scale clustering and local scale clustering. Essentially, two types of distances, i.e., Euclidean (or spatial) distance and genomic (or sequential) distance, can be used in data clustering. Clusters from sequence-based global scale models optimize spatial distances, meaning spatially adjacent loci are more likely to be assigned into the same cluster. Sequence-based local scale models, on the other hand, result in clusters that optimize genomic distances. That is to say, in these models, sequentially adjoining loci tend to be cluster together. We propose two sequence-based multiscale models (SeqMMs) for the study of chromosome hierarchical structures, including genomic compartments and topological associated domains (TADs). We find that genomic compartments are determined only by global scale information in the Hi-C data. The removal of all the local interactions within a band region as large as 10 Mb in genomic distance has almost no significant influence on the final compartment results. Further, in TAD analysis, we find that when the sequential scale is small, a tiny variation of diagonal band region in a contact map will result in a great change in the predicted TAD boundaries. When the scale value is larger than a threshold value, the TAD boundaries become very consistent. This threshold value is highly related to TAD sizes. By the comparison of our results with those previously obtained using a spectral clustering model, we find that our method is more robust and reliable. Finally, we demonstrate that almost all TAD boundaries from both clustering methods are local minimum of a TAD summation function.

## Introduction

The chromosome, the physical realization of genetic information, is one of the most complex and important cellular entities [1–7]. Over the past few decades, the significance of its three-dimensional architecture for supporting essential biological functions, such as DNA replication, transcription, repair of DNA damage and chromosome translocation, has gradually been realized [8–11]. Chromosome conformations are found to be deeply involved in the development of epigenetic organizations, the regulation of genome functions and the epigenetic inheritance of various cell states [8]. A thorough understanding of the chromosome three-dimensional structure is of fundamental importance to the decryption and interpretation of genetic information, and has become one of the most important topics in genomic and epigenetic research. Chromosome conformation capture (3C) technique [12, 13] and its derived methods, including chromosome conformation capture-on-chip (4C) [14, 15], chromosome conformation capture carbon copy (5C) [16] and high-throughput chromosome conformation capture (Hi-C) [17], have been developed and begun to uncover general features of genome organization [17–25].

Recent studies on Hi-C data have demonstrated the existence of two types of structures known as topologically associating domains (TADs) [18, 19] and genomic compartments [17]. TADs are chromosome components that are about 200 kilobases(Kb) to 2 megabases(Mb). They are originally found as the contiguous square regions along the diagonal Hi-C maps with large contact values. More importantly, TADs are very consistent between different cell types and species and their spatial distributions are highly correlated with many genomic features such as histone modifications, coordinated gene expression, lamina and DNA replication timing. Through principle component analysis, two types of genomic compartments, i.e., A and B, have been identified [17]. More specifically, the compartment B is more densely packed with higher contact frequencies. On the contrary, the compartment A is chromosome regions that are more open and accessible. It strongly correlates with the gene loci and higher gene expression. More recently, analysis on the 1Kb resolution Hi-C data indicates the existence of six different subcompartments [26].

Based on Hi-C data, various algorithms and models are proposed to study the hierarchical structure of chromosomes [17, 18, 27–35]. Since TADs are essential to the understanding of relationship between chromosome structure and gene transcription, developing efficient algorithms to detect TADs is an important topic in Hi-C data analysis. Computationally, hidden Markov models (HMMs) are the first method to identify TADs [18]. Based on the contacts located 2Mb upstream and downstream, a directionality index of a locus is calculated in this model and used to capture the sharp transitions at TADs boundaries. After that, the arrowhead algorithm with a “corner score” is proposed [26]. This special score indicates the likelihood of each locus to be at a TAD boundary and can be efficiently evaluated by using dynamic programming. Meanwhile, a resolution parameter is considered to identify TADs at various scales. This algorithm has been incorporated into the software Armatus [27]. Further, a block-wise segmentation model called HiCseg [28] is proposed. This method reduces the problem of maximizing the likelihood with respect to the block boundaries into a 1D segmentation problem, and then employ the standard dynamic programming. More recently, a spectral graph theory based model is developed for the identification of TADs [36]. In this model, Laplacian based graph segmentation is applied iteratively to obtain TADs at the given compactness level. All the above mentioned methods can be roughly divided into two categories, optimization based local models and graph based global models. In local models, TAD indicators, including directionality index, corner score, likelihood of TAD boundaries, block-segmentation, are all evaluated locally within a certain region. In global models, TAD indicators, including eigenvectors, within-cluster variance, cluster distances, among others, are all evaluated globally in the whole domain of Hi-C data.

In this paper, two sequence-based multiscale models (SeqMMs) are introduced. Unlike previous clustering models, we measure the “similarity” of loci by not only their spatial distances but also their sequential distances. With the combination of spectral graph method, we find that clusters from sequence-based global scale models optimize Euclidean distance relations, and these models can be used in genomic compartment analysis. In contrast, clusters from local scale models optimize genomic distance relations, and these models can be used in TAD analysis. Essentially, our SeqMMs provide a way to explore the hierarchical structures of chromosomes.

Mathematically, genomic compartments are defined from principal component analysis [17], they are global structural features. The loci in the same genomic compartment are spatially close to each other. But their sequential distances can be very large. Based on global scale clustering, we design Type-1 SeqMM and use it for genomic compartment analysis. In contrast, TADs are local structural features. The loci in the same TAD are not only spatially close to each other, but also sequentially adjacent to each other. Their sequential distances are usually within a certain genomic distance. Based on local scale clustering, we introduce Type-2 SeqMM and use it in TAD analysis.

## Methods

**Fig 1:**
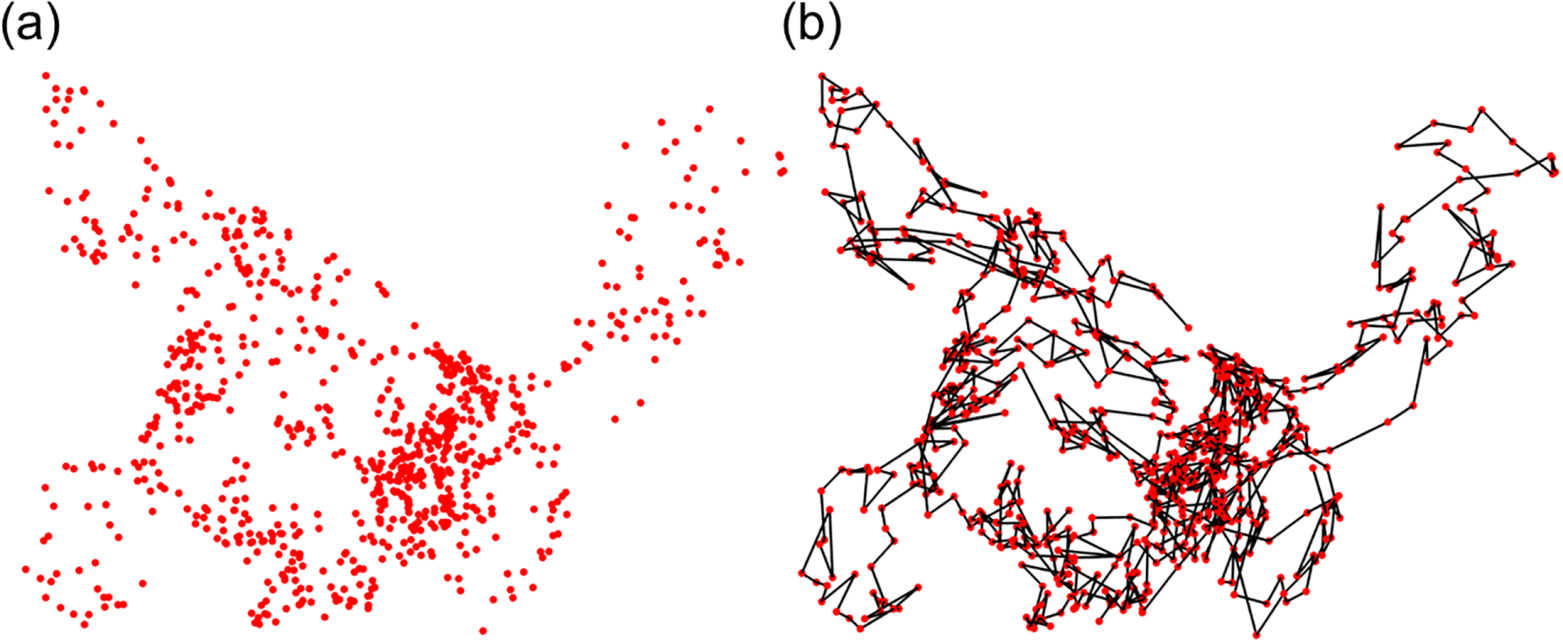
The comparison between point cloud data and biomolecular data. The simulated configuration of Human ES Cell chromosome 22 are generated by using the software shRec3D [33].

As a discrete representation of geometries, manifolds, high-dimensional structures, abstract relations and complicated subjects, point cloud data (PCD) are widely used in computer science, engineering, scientific computing and data science. Particularly, PCD and PCD based classification or clustering methods [37], including K-means, hierarchical clustering, spectral clustering, modularity, graph centrality, network approaches, etc, have been constantly used in biomolecular data analysis. However, as demonstrated in Fig 1, biomolecular structure data are essentially different from the general PCD, as they incorporate a unique sequential information. The simulated structure corresponds to chromosome 22 from Human ES Cell line and is generated by using software shRec3D [33].

**Fig 2:**
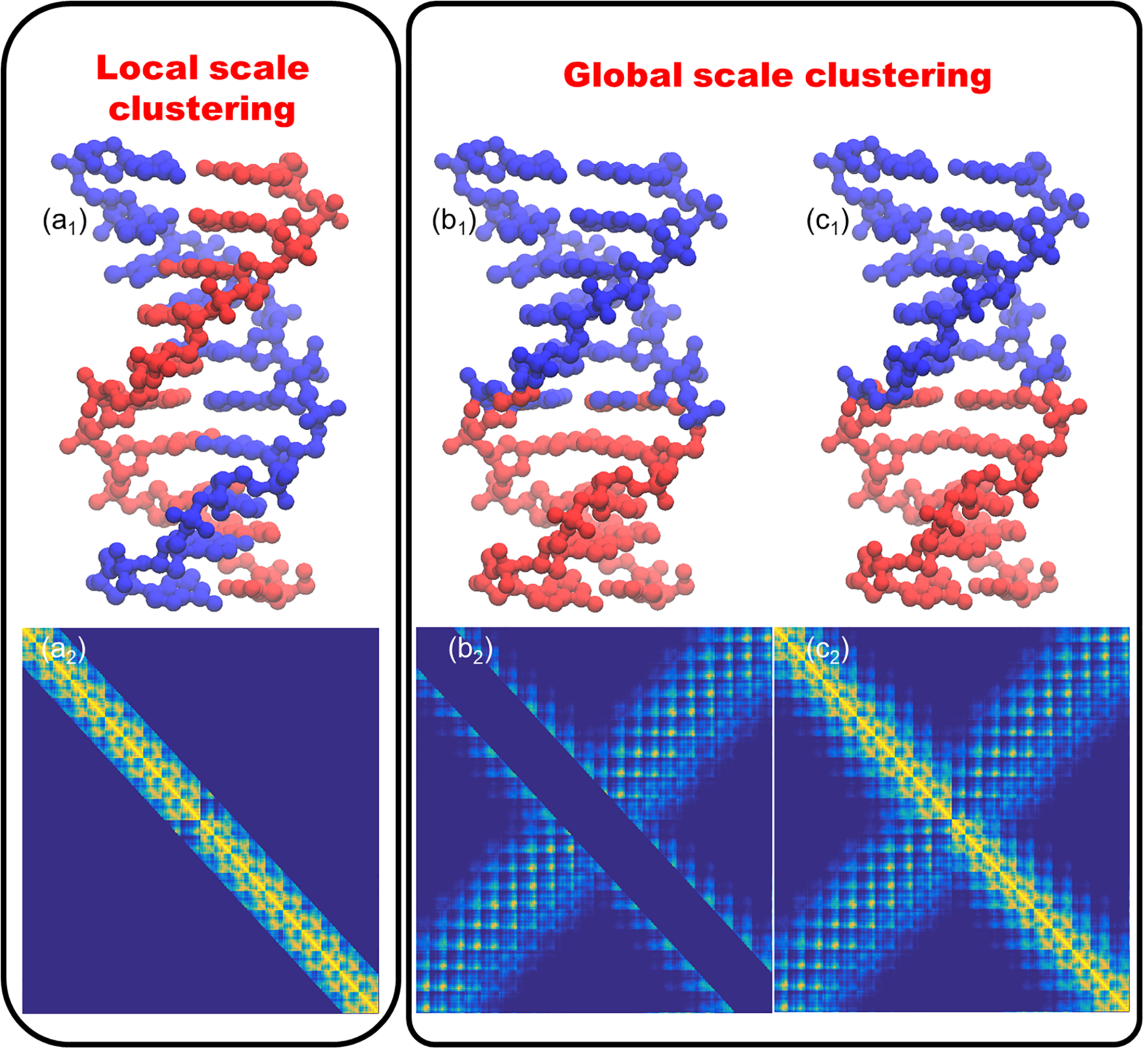
The illustration of two essential different clustering approaches, local scale clustering and global scale clustering. The local scale clustering optimizes sequential distances and is suitable for TAD analysis. The global scale clustering only considers spatial information and can be used in genomic compartment analysis.

To have an intuitive understanding of the sequential information in PCD analysis, we consider a DNA structure with PDB ID 1ZEW. Using atomic coordinates, a weight matrix is constructed. The weight values are defined by using the rigidity function [38],

$$M=\{M_{ij}=e^{-{\left(r_{ij}/\eta \right)}^{2}}|\;i=1,2,\ldots ,N;\;j=1,2,\ldots N\},$$ (1)

where *r*_*ij*_ is the Euclidean distance between *i*-th and *j*-th atoms, *N* is the total number of atoms and *η* is the scale parameter that controls the influence range of each atom. In this case, we choose *η* = 8 Å. The weight matrix is illustrated in Fig 2(**c**_2_). Two more matrices are constructed by dividing the weight matrix into a diagonal band region as in Fig 2(**a**_2_) and the remaining off-diagonal regions as in Fig 2(**b**_2_). Based on these three matrices, we can decompose the DNA structure into two parts using the spectral clustering method [37, 39]. Results are illustrated in Fig 2(**a**_1_), 2(**b**_1_) and 2(**c**_1_). It can be seen that, if we only consider relations between sequentially adjacent atoms, which are represented in the diagonal region, the DNA structure will be clustered into two complementary helix chains. However, if we use the whole matrix or only off-diagonal regions, the DNA structure will be divided in the middle region with two chains in each cluster.

Generally speaking, Fig 2 demonstrates two types of sequence-based models, i.e., sequence-based local models and sequence-based global models. It can be seen that their properties in structure decomposition differ greatly. In the first type, atoms with shorter sequential distances are more likely to be grouped into the same cluster. In the second one, spatially close atoms, i.e., atoms with large weight values, are more likely to be assigned to the same cluster. Mathematically, the sequence-based local model optimizes sequential distances, while the global model optimizes spatial distances or Euclidian distances. All PCD based classification and clustering methods belong to the second type. Therefore, the direct application of these methods in biomolecular data analysis may have some limitations.

**Fig 3:**
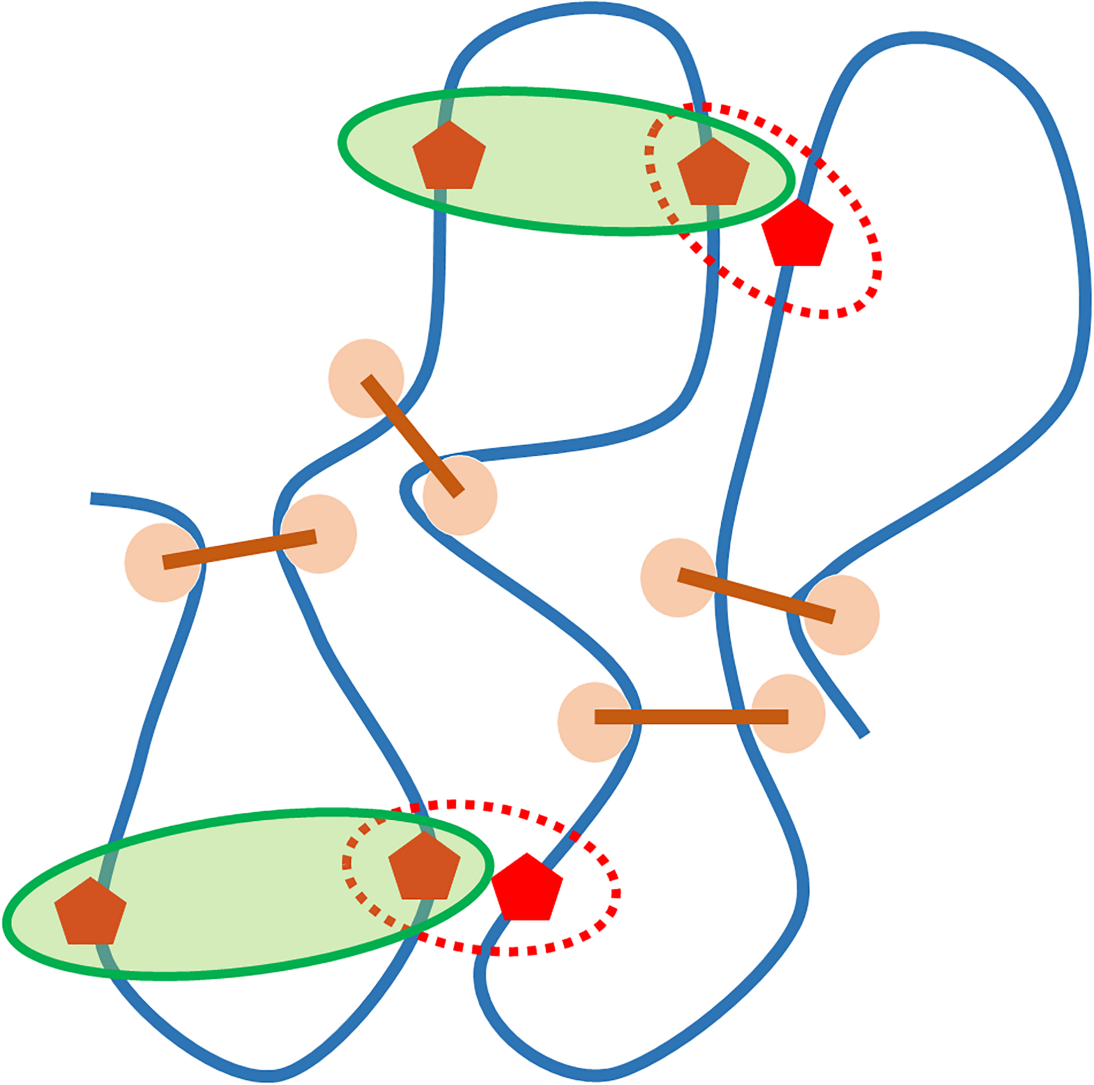
A potential problem for global scale clustering in TAD analysis. Each locus is represented by a red pentagon. Global scale clustering considers only spatial relations, thus groups loci in each red dash circle as a cluster. Biologically, loci in each green circle are more favorable to be clustered into the same TAD, as their sequential distances are much shorter. The missing of sequential information in global scale clustering will cause problems in the TAD analysis.

In Hi-C data analysis, sequential information is usually highly relevant. Fig 3 demonstrates a potential problem for global scale clustering in TAD analysis. In this figure, genomic loci are represented by red pentagons. It can be seen that the spatial distance between the two loci in any red circle is much shorter than the one in green circles, while sequential distances are exactly the opposite. If we use the traditional PCD based clustering methods, two loci in the same red circle will always have priority to be clustered into the same TAD. Obviously, this will cause serious interpretation problems, as the sequential distance between the two loci can be much larger than the size of a TAD.

### Sequence-based multiscale modeling

It should be noticed that two distance definitions, i.e., Euclidean distance and sequential distance, are greatly different and matter a lot in multiscale modeling. The Euclidean distance is just the three dimensional distance between two elements. In Hi-C data, Euclidean distance between genomic loci is inversely related to their contact frequencies [35]. In contrast, the sequential distance is defined between two elements on chains or polymers. If sequential numbers are assigned to elements, a sequential distance is just the difference between these two integers and it is always an integer. In Hi-C data, sequential distance between two loci is their genomic distance.

Even though graph and network based multiscale models are widely used in biomolecular structure and function analysis [40–49], the measurement defined in these models are in terms of the Euclidean distance. To be more specific, when we discuss atomic scale, residue scale, second structure scale, tertiary structure scale, etc, we are analyzing structural elements based on their sizes measured in Euclidean distances.

In this section, the sequence-based multiscale modeling is proposed for biomolecular data analysis, particularly for Hi-C data analysis. In our multiscale models, a scale parameter *N*_*b*_ is defined not from the Euclidean distance but from the sequential distance. The parameter *N*_*b*_ can be viewed as a cut-off sequential distance. In Hi-C matrices, the parameter *N*_*b*_ specifies the size of the diagonal band region. Further, two sequence-based multiscale models are proposed for analyzing chromosome genomic compartments and TADs. These two models, denoted as Type-1 SeqMM and Type-2 SeqMM, are derived from the perspective of local scale clustering and global scale clustering, respectively.

#### Type-1 SeqMM

In Type-1 SeqMM, we remove sequentially short-range interactions by changing the value of scale parameter *N*_*b*_. More specifically, for a Hi-C matrix, a diagonal band region with size *N*_*b*_ is systematically removed from the model, resulting a new Hi-C matrix as following,

$$\begin{array}{c} M_{ij}^{\mathrm{Seq}}=\{\begin{array}{c} \begin{array}{cc} M_{ij}, & \left|i-j\right|\geq N_{b}\\ 0, & |i-j|<N_{b} \end{array}. \end{array} \end{array}$$ (2)

Here *M*_*ij*_ can be the original or normalized contact frequencies. It can seen that our Type-1 SeqMM is defined by taking away the local interactions from the model and is designed for global scale clustering. An example can be found in Fig 6(**a**) to 6(**c**). We suggest that it can be used in chromosome genomic compartment analysis.

#### Type-2 SeqMM

In Type-1 SeqMM, when short-range interactions are systematically removed from the biomolecular data, long-range interactions are preserved. Type-2 SeqMM is designed in the exact opposite way,

$$\begin{array}{c} M_{ij}^{\mathrm{Seq}}=\{\begin{array}{c} \begin{array}{cc} M_{ij}, & \left|i-j|>0\;\mathrm{and}\;|i-j\right|⩽N_{b}\\ 0, & |i-j|>N_{b}\\ -\sum_{i\neq j}^{N}M_{ij}^{\mathrm{Seq}}, & i=j \end{array}. \end{array} \end{array}$$ (3)

The scale parameter *N*_*b*_ controls the size of the diagonal band region.

Mathematically, our SeqMM matrix in Eq (3) is a weighted Laplacian matrix, which plays an important role in graph representation and spectral clustering [37, 39, 50]. The second smallest eigenvalue and its associated eigenvector from the Laplacian matrix, are known as the Fiedler value (or algebraic connectivity) and the Fiedler vector, respectively. The Fiedler value is an important measurement of the general topological connectivity of a graph. The Fiedler vector gives an optimized classification of a graph into two separated domains [39, 50]. In our Type-2 SeqMM, the local interaction region can be systematically enlarged to model the different scales of interactions.

Type-2 SeqMM is proposed for chromosome TAD analysis. After Hi-C data preprocessing, a weighted Laplacian matrix can be generated by using a suitable scale value *N*_*b*_. The TAD number in the data is estimated based on size and resolution of the Hi-C matrix. We assume the size of TAD to be around 2Mb, and TAD number *N*_*c*_ can be roughly calculated by dividing the total length over 2Mb. The basic procedure is presented in **Algorithm 1**. It should be noticed that the final number of TADs is usually larger than *N*_*c*_. The Code is available at S1 File.

**Algorithm 1** Type-2 SeqMM for TAD analysis

**Pre-processing**: Remove all rows and columns, that summed together equal to zero (or smaller than a pre-defined range); Transform the Hi-C contact frequencies to spatial distances (default function *f*(*x*) = *log*(1 + *x*));

**Step 1**: Choose a scale parameter *N*_*b*_ to construct a weighted Laplacian matrix as in Eq 3;

**Step 2**: Calculate the first *N*_*c*_ eigenvectors. Here *N*_*c*_ is the estimated number of TADs;

**Step 3**: Employ the K-means algorithm on the *N*_*c*_ eigenvectors to identify *N*_*c*_ clusters;

**Step 4**: Subdivide each cluster into several TADs until the loci in each TAD are sequentially contiguous.

## Results

### Genomic compartments

The genomic compartment is defined from the principal component analysis of Hi-C data. Mathematically, the principal component captures the global shape of a structure. In Chen’s spectral method [36], it shows that the genomic compartment results from the first principal component (FPC) are identical to the predictions made from the lowest-frequency eigenvector of weighted Laplacian matrices. More interesting, as proved in the elastic network model and normal mode analysis, these lowest-frequency eigenvectors are uniquely determined by the global geometric information of structures [51–54].

**Fig 4:**
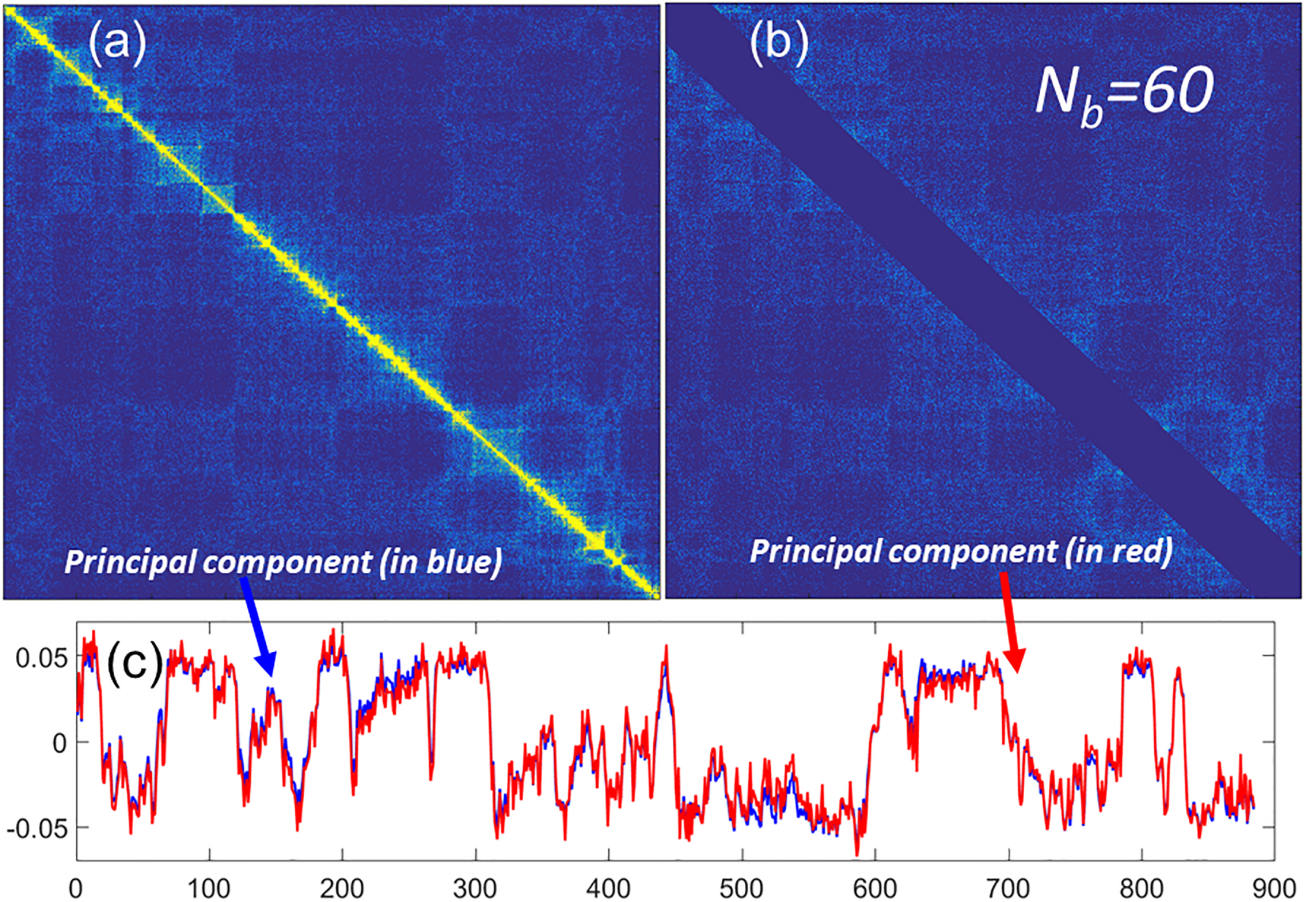
The irrelevance of local interactions in genomic compartment analysis. (**a**) The 100Kb resolution Hi-C data for GM06990 chromosome 14. (**b**) The chromosome 14 Hi-C data with zero contact frequencies in the diagonal band region. The band size, or scale parameter, *N*_*b*_ equal to 60, which amounts to 6 Mb genomic distance. (**c**) The principal components from the two matrices, blue line for (**a**) and red line for (**b**), have almost the same behavior. The Pearson correlation coefficient between these two first principal components is 0.991.

Since the FPC describes the global properties of a structure, we use the Type-1 SeqMM for our genomic compartment analysis. We consider the GM06990 chromosome 14 data with resolution 100Kb. This is a classic example used for genomic compartment analysis [17]. Before the principal component analysis (PCA), the chromosome 14 Hi-C matrix is processed. We remove all columns and rows with all zero values and normalize the matrix using the Toeplitz matrix [17]. After that, we construct a new matrix by removing the diagonal band region with *N*_*b*_ = 60 from the normalized Hi-C matrix, and calculate its FPC. Further, we compare this new FPC with the original one. Results are shown in Fig 4. The blue line represents the FPC from the original Hi-C matrix and red line represents the FPC from the off-diagonal matrix. It can be seen that they are almost identical to each other. Actually, the Pearson correlation coefficient (PCC) between the two FPCs is as high as 0.991, meaning that the removal of the diagonal band region have almost no influence to the FPC.

**Fig 5:**
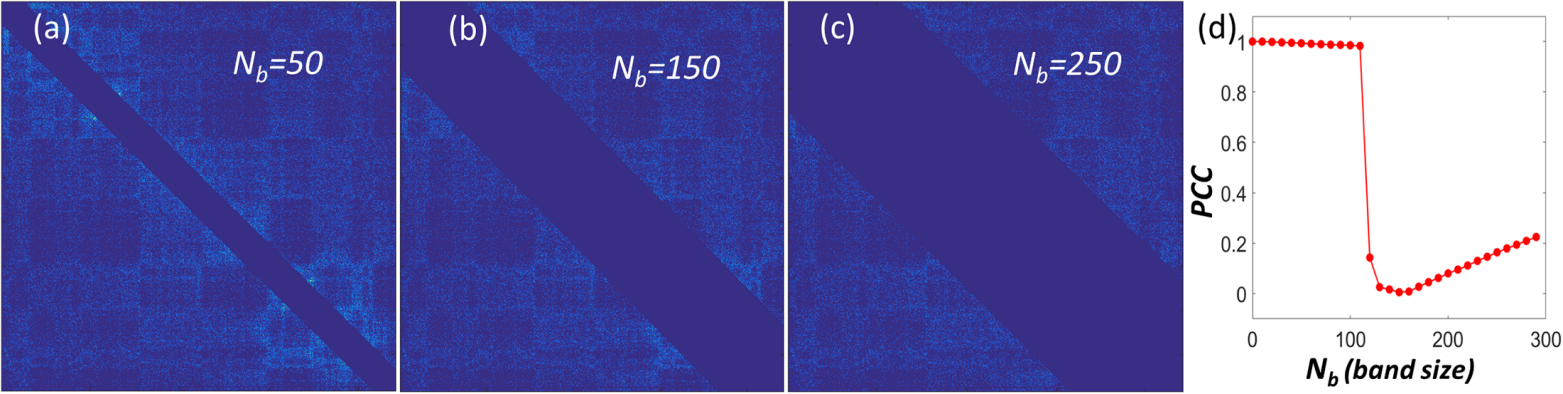
The Type-1 SeqMM for genomic compartment analysis of GM06990 chromosome 14. It is found that the local interactions within a band size around 10 Mb genomic distance contribute very little to genomic compartments. (**a**)-(**c**) The illustration of the Hi-C matrices in Type-1 SeqMM. The sizes of the diagonal band region removed from the Hi-C data go from *N*_*b*_ = 50, *N*_*b*_ = 150 to *N*_*b*_ = 250. (**d**) The PCCs between the first principal components from the original Hi-C matrix and the Hi-C matrices from our Type-1 SeqMM. A high PCC value is observed when the band size is smaller than 10 Mb, meaning the removal of data in this band region has almost no significant influence in the genomic compartments.

To have a more quantitative understanding of the FPC and Hi-C diagonal regions, we continuously change the value of the scale parameter *N*_*b*_ to generate a series of Hi-C matrices at different scales. Then we systematically calculate their FPCs and measure the similarity between these FPCs with the original one by PCCs. Results are shown in Fig 5. It can be seen that PCCs changes very slowly when scale parameter is smaller than 100, which is 10 Mb in genomic distance. State differently, we can get almost the same genomic compartment even when we remove all the Hi-C data within the 10 Mb band region. It should be noticed that almost all the largest Hi-C value, i.e., contact frequencies, are located within this 10 Mb band. These values, however, are irrelevant to the chromosome genomic compartment!

**Fig 6:**
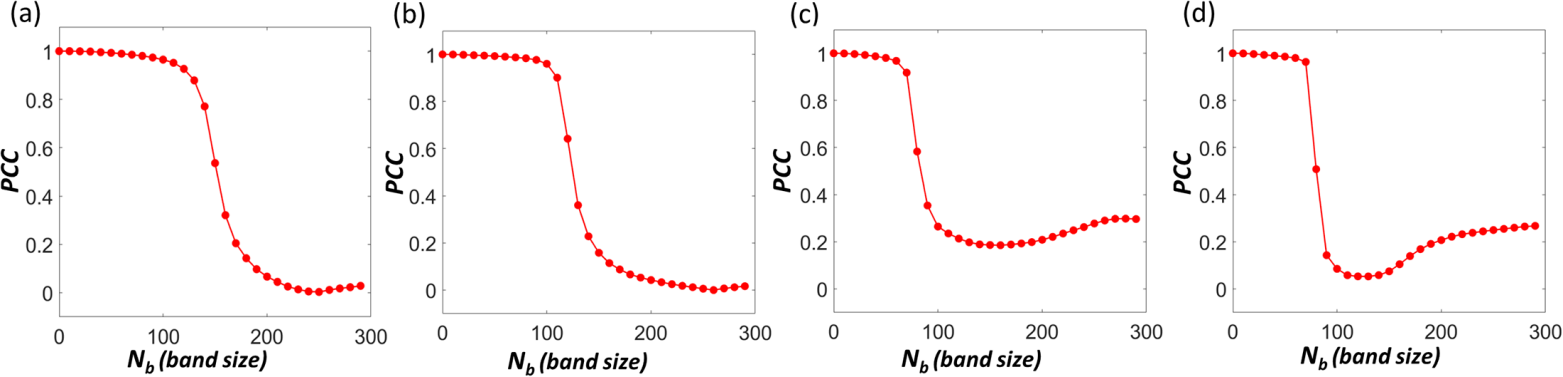
The Type-1 SeqMM for genomic compartment analysis of GM06990 chromosomes 1, 5, 9 and 13. (**a**)-(**d**) The PCCs between the first principal components from the original Hi-C matrix and the Hi-C matrices from our Type-1 SeqMM. Form (**a**) to (**d**), the PCC results are for GM06990 chromosomes 1, 5, 9 and 13, respectively. A consistent behavior has been observed. High PCCs are obtained when band sizes are smaller than around 10 Mb genomic distance. The results confirm our finding that local interactions within a special band region have very little contribution to genomic compartments.

We further test our SeqMM on other GM06990 chromosomes. A very consistent pattern can be observed. Results of chromosomes 1, 5, 9 and 13 are illustrated in Fig 6. It can be seen that the shape decrease of PCCs is usually found at around 100 locus (10 Mb in genomic distance). This indicates a transition between local scales to global scales. Further studies are needed to explain its biological implications.

### Topological associated domain

Another very important finding in Hi-C data analysis is the topological associated domain. TADs are megabase-sized local chromatin interaction domains. They have loop structures and are highly stable and conserved across various cell types and species. TAD boundaries are found to be enriched with the protein CTCF, housekeeping genes, transfer RNAs and short interspersed element (SINE) retrotransposons [18, 23, 24, 26]. These components play important roles in establishing and supporting TADs and other architectural structures of the chromosome. Due to the structural and functional importance of TADs, various algorithms have been proposed for the identification of TADs as stated in the introduction part. However, the sequential information is not considered in any of these models.

**Fig 7:**
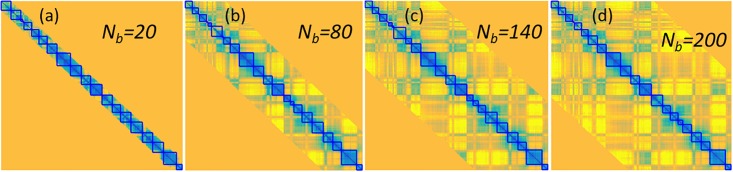
The illustration of TADs calculated for our Type-2 SeqMM. The 100Kb resolution Hi-C matrix for chromosome 22 from IMR90 cell line is considered. All predicted TAD boundaries are marked by blue lines. From **(a)** to **(d)**, the band sizes are *N*_*b*_ = 20, 80, 140 and 200, respectively. The predicted TAD boundaries are relatively consistent when *N*_*b*_ is larger than 80. To facilitate a better visualization, the matrices values are correlation coefficients of the normalized Hi-C matrix [17].

In this section, Type-2 SeqMM is used to study chromosome TADs. In our Type-2 SeqMM, the clustering is done by using K-means method on eigenvectors from spectral graph model. The basic procedure of the algorithm is illustrated in **Algorithm 1**. To explore the relation between the band size and TAD boundaries from the clustering, we consider a 100Kb resolution Hi-C matrix for chromosome 22 from IMR90 cell line. We systematically change the band size *N*_*b*_ from 20, 80, 140 to 200. The corresponding TAD boundaries are illustrated in Fig 7. It can be seen that the TAD regions evaluated from different scales are not exactly the same and have some variations. Particularly when the band size *N*_*b*_ change from 20 to 80, the calculated TAD regions are quite different. Further, when the band size is larger than 80, although the TAD boundaries are still not the same, they share more and more common values.

**Fig 8:**
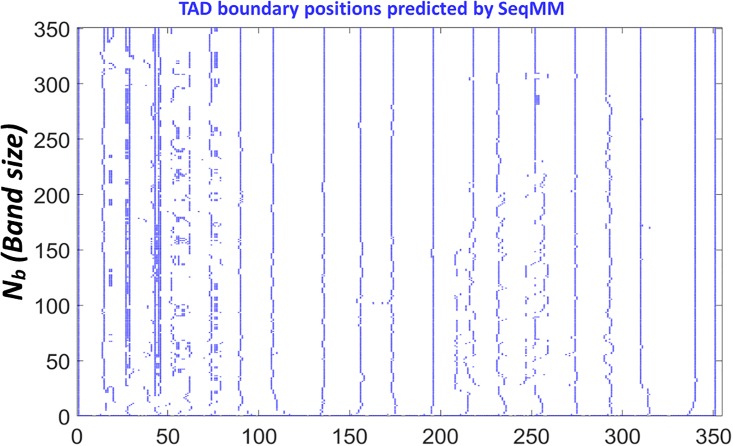
The illustration of TAD boundaries calculated by our Type-2 SeqMM. The 100Kb resolution Hi-C matrix for chromosome 22 from IMR90 cell line is considered. All predicted TAD boundaries are marked by the blue color. It can be seen that, the predicted TAD boundaries are relatively consistent.

To have a more quantitative understanding of this, we systematically change the scale parameter *N*_*b*_ from 2 to 351 (the size of the normalized Hi-C matrix) and calculate the TAD boundaries. Results are shown in Fig 8. We can find that when the value of scale parameter *N*_*b*_ is small, a tiny change of its value can result in huge variations of the predicted TAD boundaries. However, when the scale parameter is larger than a certain value, the fluctuations in the predicted TAD boundaries are greatly reduced. The threshold value is roughly about 20, which is 2 Mb in genomic distance.

**Fig 9:**
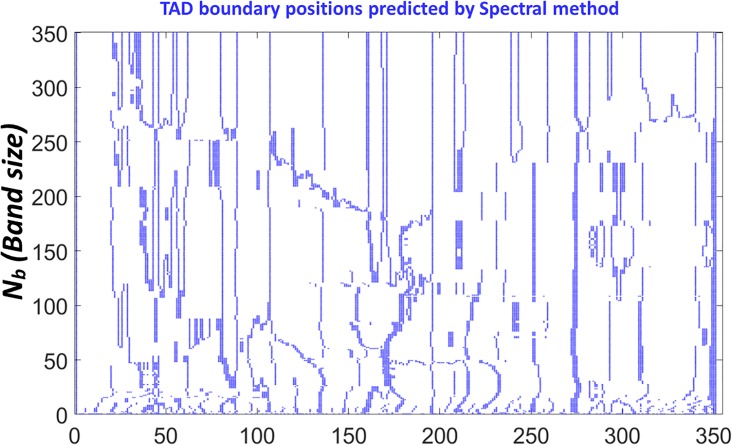
The illustration of TAD boundaries calculated by Chen’s spectral method [36] for our Type-2 sequence-based Hi-C matrix models. The 100Kb resolution Hi-C matrix for chromosome 22 from IMR90 cell line is considered. All predicted TAD boundaries are marked by the blue color. It can be seen that, the predicted TAD boundaries have a much larger variation compared with our results in Fig 8.

We further apply the spectral approach used in Chen’s method [36] on the multiscale Laplacian matrices in Eq (3). Results are shown in Fig 9. It can be seen that the variation of the predicted TAD boundaries by his method is much larger than that of our Type-2 SeqMM. More interestingly, the amplitude of variation below the threshold (2 Mb) is much larger than the one after the threshold, which is the same as in our model. Biologically, the threshold value should be highly related to the TAD properties. This is because when the band sizes *N*_*b*_ of our multiscale matrices are smaller than the size of TADs, local interactions within TADs are removed from our models, resulting in a much larger variation in predicted TAD boundaries. However, when the band size is larger than 2Mb, all TAD-related local interactions will be considered, thus a much consistent TAD boundaries should be expected. Stated differently, since TADs are mainly determined by local interactions within the 2Mb band region, the calculated TAD boundaries should always be the same for multiscale matrices with *N*_*b*_ larger than 2Mb. In this sense, our Type-2 SeqMM is much more robust and reliable than Chen’s method [36] as a much smaller variation is observed in our model when *N*_*b*_ is larger than 2Mb. Mathematically, in Chen’s spectral method, the global scale clustering is iteratively used to subdivide contact matrix or matrix region into two subregions until the algebraic connectivities within the submatrices are all smaller than certain threshold. Therefore, this method optimizes only spatial distances between different loci.

**Fig 10:**
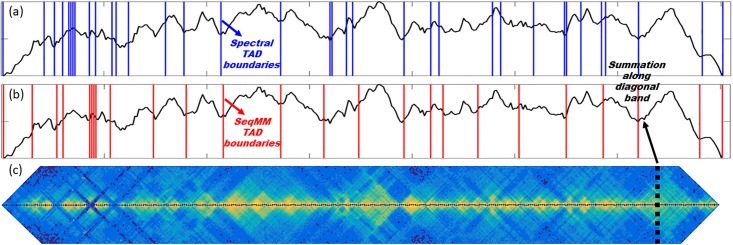
The comparison of the predicted TAD boundaries from Chen’s spectral method [36] (blue lines) and our Type-2 SeqMM (red lines). Even though the predicted TAD boundaries from two methods vary a lot, almost all of them are local minimal values of a TAD summation function. **(a)** The blue lines are TAD boundaries calculated from Chen’s method [36]. **(b)** The red lines are TAD boundaries calculated from our Type-2 SeqMM with band size 6Mb. **(c)** The diagonal band region from the normalized Hi-C matrix. Again the band size is 6 Mb. We summarize the Hi-C values along the direction that is perpendicular to the matrix diagonal, as indicated by the black dash line. The summation results are represented by the black lines in both **(a)** and **(b)**.

Further, even with the difference between the two models, both methods capture the local minimum of a TAD summation function. We consider the 100Kb resolution Hi-C matrix for chromosome 22 from IMR90 cell line. The band size *N*_*b*_ is chosen as 60, which is amount to 6 Mb in genomic sequence. We summarize the contact matric values along the direction that is perpendicular to the matrix diagonal. Results are shown as the black lines in Fig 10(**a**) and 10(**b**). The TAD boundaries from Chen’s method and our Type-2 SeqMM are illustrated by blue and red lines. It can be seen that nearly all of these lines are located at the local minima of the summation function. More interestingly, the two methods share many common TAD boundaries. This indicates that the situation illustrated in Fig 3 does not widely exist. This can also be confirmed from the behavior of off-diagonal values. Usually, the off-diagonal values decrease very quickly outside the TAD regions, meaning the distance between a locus from a TAD and a locus outside this TAD is usually very large.

## Conclusion

In this paper, we discuss a sequence-based multiscale clustering model for biomolecular data analysis. Biomolecules and their complexes are hierarchical structures made from one or several polymer chains. With the sequential information embedded in these polymer chains, biomolecular data are fundamentally different from the general point cloud data. Traditional clustering methods derived from point cloud data, fall short when sequential information matters. To overcome this problem, we propose a sequence-based multiscale model for biomolecular structure analysis. We generate a series of structural matrices by gradually and systematically removing the short-range or long-range interactions. These new matrices focus on different sequential scales and their clustering has different biological interpretations. Two SeqMMs have been applied to Hi-C data analysis. We find that the genomic compartments only relate to the global scale information. The removal of a diagonal band region as large as 10 Mb has very little influence to the finally compartment results. Further, we study TADs with our local scale models. We find that when sequence scale is small, a tiny variation of its value will result in great changes in TAD boundaries. However, when the scale value is larger than a threshold value, the TAD boundaries become very consistent. This threshold value is highly related to the sizes of TADs. Interestingly, our method is much more robust than a previous spectral clustering method in the TAD analysis.

## Supporting information

S1 File (ZIP)
